# Seed release by a serotinous pine in the absence of fire: implications for invasion into temperate regions

**DOI:** 10.1093/aobpla/plz077

**Published:** 2019-11-26

**Authors:** Sarah V Wyse, Jerusha E Brown, Philip E Hulme

**Affiliations:** Bio-Protection Research Centre, Lincoln University, Lincoln, Canterbury, New Zealand

**Keywords:** Alien, biological invasions, climate change, closed-cone pine, conifer, intraspecific trait variability, *Pinus radiata*, weed

## Abstract

In pines, the release of seeds from serotinous cones is primarily considered a response to the high temperatures of a fire. However, the naturalization of serotinous pines in regions where fires are rare highlights the need to quantify environmental conditions that determine seed release to allow accurate prediction of dispersal and spread risk. We investigated the conditions that break cone serotiny in *Pinus radiata*, a widely planted forestry species that has naturalized in temperate regions worldwide. We quantified the cone temperatures at which cones open in this species, while also assessing potential confounding effects of cone moisture and age on these temperature requirements. We compared our laboratory results with cone opening behaviour under typical field conditions during summer in Canterbury, New Zealand. Cones opened at a mean temperature of 45 °C, much higher than maximum ambient air temperatures recorded in New Zealand. We found no influence of cone age or moisture content on opening temperature. Under field conditions, cones opened upon reaching similar temperatures to those determined in the laboratory; however, passive solar heating caused cones to reach temperatures up to 15 °C higher than ambient conditions. This resulted in 50 % of cones opening in field conditions where maximum air temperatures never exceeded 30 °C. Our results highlight the need for complementary laboratory and field experiments for understanding seed release from serotinous cones. Our findings have important implications for weed risk assessments, showing that serotinous pines can release seed in temperate climates without fire.

## Teaser

Serotiny is a plant trait generally associated with fire, and it is often thought that this trait may prevent serotinous pine species becoming invasive in habitats where fires are rare. However, this study found that cones of *Pinus radiata* will open at ~45 °C, and that heating by the sun allows cones to reach these temperatures when ambient air temperatures are only 25–30 °C. The results have important implications for weed risk assessments, showing that serotinous pines can release seed and disperse in temperate climates without the aid of fire.

## Introduction

Serotinous cones retain their seeds for one or more years following maturity (Critchfield 1957; Lamont 1991), with seed release often occurring in response to an environmental stimulus such as high temperature. Within the genus *Pinus* (pines hereafter) the retention of seeds in serotinous cones is widely understood to reflect selection in fire-prone environments (Schwilk and Ackerly 2001; Hernández-Serrano *et al.* 2014; Pausas 2015; Badik *et al.* 2018). Seeds may be retained in the tree canopy for between 3 (*P. halepensis*) and 50 years (*P. pinaster*) until the heat of a fire acts to break the resin bonds holding the cone scales closed, simultaneously releasing seeds into the environment (Baskin and Baskin 2014). Over several years, serotinous pines may build up large canopy seed banks during years favourable for reproduction and then, following a fire, synchronously disperse seeds into a nutrient rich, low competition environment in sufficient numbers to satiate seed and seedling predators (Lamont 1991). Consequently, the occurrence of serotinous cones has been viewed as an important pre-adaptation that has facilitated the widespread invasion of pine species into fire-prone environments of the Southern Hemisphere (Franzese and Raffaele 2017).

However, pines have also been introduced to regions outside their native climate niche, particularly to regions with cooler, wetter and less seasonal climates (Essl *et al.* 2011; Perret *et al.* 2019). Such environments are often described as non-fire-prone (*sensu*Lamont and He 2017) and are typically characterized by plant species without unambiguous adaptations to fire (i.e. Richardson *et al.* 2015). In oceanic or temperate climates where fire is less frequent it is possible that the retention of seeds in serotinous cones may impede rather than facilitate pine invasion. In a global analysis of pine naturalization, serotiny explained little variation in success among a wide range of pine species, which might reflect its importance under some but not all climates (Procheş *et al.* 2012). However, in a detailed analysis of pine naturalization in non-fire-prone habitats in the Northern (UK) and Southern (New Zealand) Hemispheres, serotiny was not found to be an important trait; neither facilitating nor impeding establishment (McGregor *et al.* 2012). The lack of influence of serotiny on pine naturalization success may reflect the considerable intraspecific variation that exists in some pine species with respect to the degree to which trees retain seeds in their canopies. Within a species, the proportion of serotinous cones in the canopy may vary with tree age and size, environmental conditions such as stand density and fire frequency, as well as genetic provenance (Baskin and Baskin 2014; Martín-Sanz *et al.* 2016). Thus, while *Pinus contorta* ssp. *latifolia* is often classed as serotinous, in the cooler, wetter climate of Sweden up to 70 % of trees in commercial plantations bear non-serotinous cones (Despain 2001). As a result, this species has naturalized widely in Sweden (Sykes 2001). It is therefore evident that, for *P. contorta*, serotiny of the local forestry genetic pool is the variable of interest, rather than a single classification for the species as a whole. Additionally in some species, seeds may be released from serotinous cones in the absence of fire, such as following particularly hot or dry weather conditions (Keeley and Zedler 1998). Yet whether this is also a function of cone age or cone moisture content remains unclear.

Although considerable data exist detailing the pine species that can produce serotinous cones, these data are, by necessity, qualitative, and the presence of serotiny in a species is usually treated as a binary or ternary (serotinous, moderately serotinous or not serotinous) trait (Richardson *et al.* 1990; McGregor *et al.* 2012; Procheş *et al.* 2012). However, understanding the behaviour of serotinous cones with respect to environmental stimuli, as well as the within- and among-species variation in such traits, is key for understanding the spread risk of pines when introduced to novel environments, and predicting both the timing of seed release and the associated quantity of seeds that may enter the environment. For most pines there has been a dearth of work examining the suite of conditions that can lead to seed release from serotinous cones, yet a variety of methodological approaches have been applied where these conditions have been examined. Typical approaches focus on the temperatures required to release the scales of serotinous cones, and involve either heating cones in ovens or by immersion in water (Beaufait 1960; Perry and Lotan 1977; Tapias *et al.* 2001; Reyes and Casal 2002). Cone desiccation, without heating, has also been examined for *P. halepensis* (Martín-Sanz *et al.* 2017), yet the potential confounding effects of temperature and desiccation have not been examined.

Here, we investigate the conditions that lead to the breaking of serotiny in *Pinus radiata* cones, a species planted abundantly throughout the Southern Hemisphere. In the laboratory, we assess the conditions required to release the scales of the serotinous cones by disentangling the direct effect of heat from of the indirect effect of drying cones, both of which may independently lead to cone opening. Importantly, these laboratory-based estimates are then compared to cone opening behaviour in the field under natural weather conditions in Canterbury, New Zealand. As a result, we hope to encourage more detailed measurement of the environmental determinants of seed release from serotinous cones within *Pinus* to allow prediction of the timing and likelihood of seed dispersal in novel environments or under changing climate conditions.

## Materials and Methods

### Study species

*Pinus radiata* (Monterey pine, radiata pine) is the world’s most extensively planted softwood (Mead 2013). The species has generally been treated as being fully serotinous (Richardson *et al.* 1990; McGregor *et al.* 2012; Procheş *et al.* 2012), and cones can remain closed in the canopy for over 20 years (Baskin and Baskin 2014). In the native range in California, USA, fire provides the optimal conditions for *P. radiata* cones to open, but cones may also open in hot dry weather (Rogers 2002). Due to the incidence of cool humid conditions in the native range, cone opening in the absence of fire is a process that occurs infrequently in these habitats (but see Keeley and Zedler 1998). The few attempts to examine the temperature conditions required to allow cones to open in the absence of fire have addressed temperatures far in excess of ambient conditions (100–500 °C; Reyes and Casal 2002). Compared to the native range, New Zealand has similar average summer temperatures, although precipitation during the summer months is much higher in New Zealand climates (Fick and Hijmans 2017) and forest fires are both less frequent, and smaller in scale (Perry *et al.* 2014). Therefore, the marked serotinous habit has led to *P. radiata* being perceived as having a low risk of establishment outside of cultivation in New Zealand (Froude 2011), despite the species escaping cultivation to become naturalized, and even invasive, in other temperate climate regions worldwide (Essl *et al.* 2010).

### Opening temperature of *Pinus radiata* cones

In the laboratory, we assessed the temperature at which serotiny was broken for 100 closed, ripe *P. radiata* cones collected from 10 trees in lowland Canterbury, New Zealand. The *P. radiata* commonly grown in New Zealand descend predominantly from the Monterey and Año Nuevo populations, and although bred for forestry are still thought to be genetically close to the native provenances (Burdon *et al.* 2008; Mead 2013). Trees selected for sampling were all planted, similarly-sized, mature individuals either from shelterbelts (i.e. windbreaks) or small plantation stands. Two methods were used to measure the opening temperatures of these cones, with five cones from each tree randomly assigned to each method: heating in a water bath, or a laboratory oven. Both methods involved the gradual increase of cone temperature until the point at which the scales released. *Pinus radiata* cones are initially ‘shiny light brown’ in colour upon maturity, before weathering to a ‘dull grey’ in subsequent seasons (Farjon 2005). Given that cone age has been found to influence opening temperature in some species, such as *P. halepensis* (Tapias *et al.* 2001), we used cone colour as a proxy for age to compare cones from the current season with older closed cones that have been retained for one or more seasons. This allowed us to ensure any patterns found were not the result of confounding effects from cone aging.

Although not simulating natural conditions experienced by cones on a tree, the water bath method is a commonly used technique that directly assesses the effect of temperature on cone opening, and has been used to measure cone opening temperature of serotinous pines such as *P. contorta* (Perry and Lotan 1977), *P. halepensis* and *P. pinaster* (Tapias *et al.* 2001). Here, we submerged the cones in a glass beaker containing 500 mL of water at room temperature (23 °C), which was then placed in a Precitherm PFV water bath (Boehringer Mannheim) heated to 60 °C. Water temperature in the beaker was continuously monitored with a thermometer and reached equilibrium with the surrounding water in ~10 min. As has been observed in other pines, scale release in *P. radiata* is abrupt, and generally associated with an audible crack. Perry and Lotan (1977) defined cone-scale opening as the point at which at least one scale on the cone had broken its resin bond and released; however, here we considered a cone with 15 % of its scales released to be ‘open’. Generally, scale release was reasonably synchronous with at least 50 % releasing at once.

Given that dry conditions have been described as aiding cone opening in serotinous pines, such as *P. halepensis* (Nathan *et al.* 1999), we also assessed the temperatures required to break cone serotiny in a dry environment. This method was more reflective of natural conditions experienced by a cone if cone temperature were in equilibrium with the ambient air temperature, but allows the potentially confounding effects of cone drying and cone heating. Cones were placed in a laboratory oven (MLS, Contherm Scientific Company) at 24 °C, and oven temperature was increased by ~2 °C every 24 h until all cones had opened (52 °C). Internal oven temperature was monitored with a thermometer, and every cone was inspected after each 24-h period to assess the percentage of the scales that had released. As with the water bath method, an open cone was defined as a cone with 15 % of the scales released, although again scale release was generally reasonably synchronous. On average, moisture content of cones upon scale release in the oven was ~40 % of the moisture content of cones recorded at room temperature (24 °C), where initial cone moisture content and moisture content upon opening were calculated on a dry mass basis (i.e. [mass at time X − dry mass]/dry mass).

Using the *lmer* function from the R package *lme4* (Bates *et al.* 2015), we performed a linear mixed model to compare the temperatures required to break serotiny between cones opened in the oven and water bath, assess the potential influence of cone age on opening temperature and investigate any potential interaction between these two variables (i.e. opening method, cone colour and their interaction as fixed effects). The identity of the parent tree was used as a random variable. Analyses were conducted in R version 3.5.0 (R Core Team 2018).

### Assessment of passive solar heating in *Pinus radiata* cones

**Figure 1.**
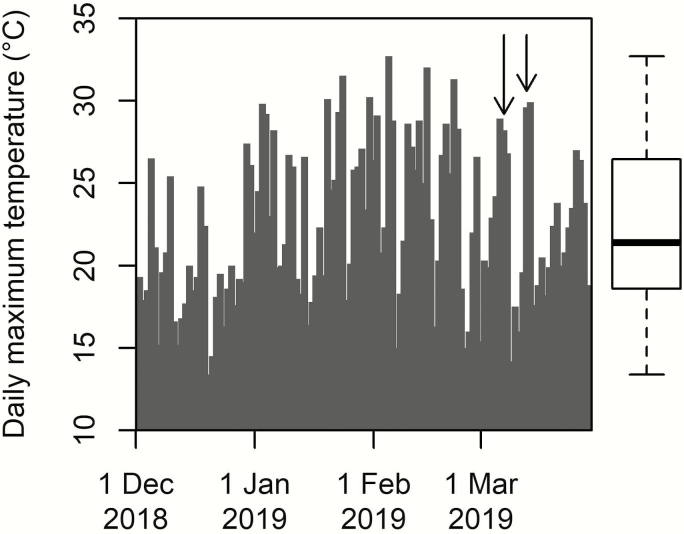
The daily maximum temperatures for the 2018/19 Southern Hemisphere summer in Lincoln, Canterbury, New Zealand. Arrows indicate the 2 days on which the experiments were undertaken. Boxplot describes the distribution of daily maximum temperature values during the 4-month period, depicting the range, interquartile range and the median value.

To assess the potential for a role of passive solar heating in cone opening, we collected a further 50 cones from 10 *P. radiata* trees in lowland Canterbury, New Zealand. On two cloudless days representative of hot summer temperatures in Canterbury (Fig. 1), we arranged five cones from each of five of the 10 trees on a wire rack 1 m above the ground in a position that received full sun all day from ~0930 h. The arrangement of cones on the wire rack allowed full circulation of air around each cone. Different cones were used on the 2 days, and all had 1.5 mm diameter and ~7 mm deep holes drilled under two cone scales to allow a thermocouple to be inserted under the scale. Every 30 min, from 0900 to 1600 h, we recorded the ambient temperature around the cones and temperature under the cone scales using a Fluke 53 II B thermometer with a Fluke model 80PK-1 Type-K bead probe thermocouple (Fluke Corporation, Everett, WA, USA). We also recorded the time and temperature under the cone scales when any cone opened. The cones that failed to open in the sun were subsequently opened in the oven to determine the temperatures required to break their serotiny. The daily maximum temperatures recorded by a local weather station ~2 km from the study location (NIWA 2019) were 28.2 °C on the first day and 29.6 °C on the second. Wind conditions differed between the 2 days: wind gusts reached a maximum of 66 km h^−1^ in the mid-afternoon on Day 1, whilst maximum wind gust speeds on Day 2 only reached 34 km h^−1^ (NIWA 2019).

We calculated cone temperature for any given time as the mean of the temperatures measured under the two scales and investigated the relationship between cone temperature and ambient temperature using a linear mixed model. Day was used as a fixed variable, with tree and cone as random variables. A linear mixed model, with day as a fixed variable and the identity of the parent tree as a random variable, was also used to compare the opening temperatures between cones that opened naturally in the sun with those that had to be opened subsequently in the oven.

## Results

### Opening temperature of *Pinus radiata* cones

**Figure 2.**
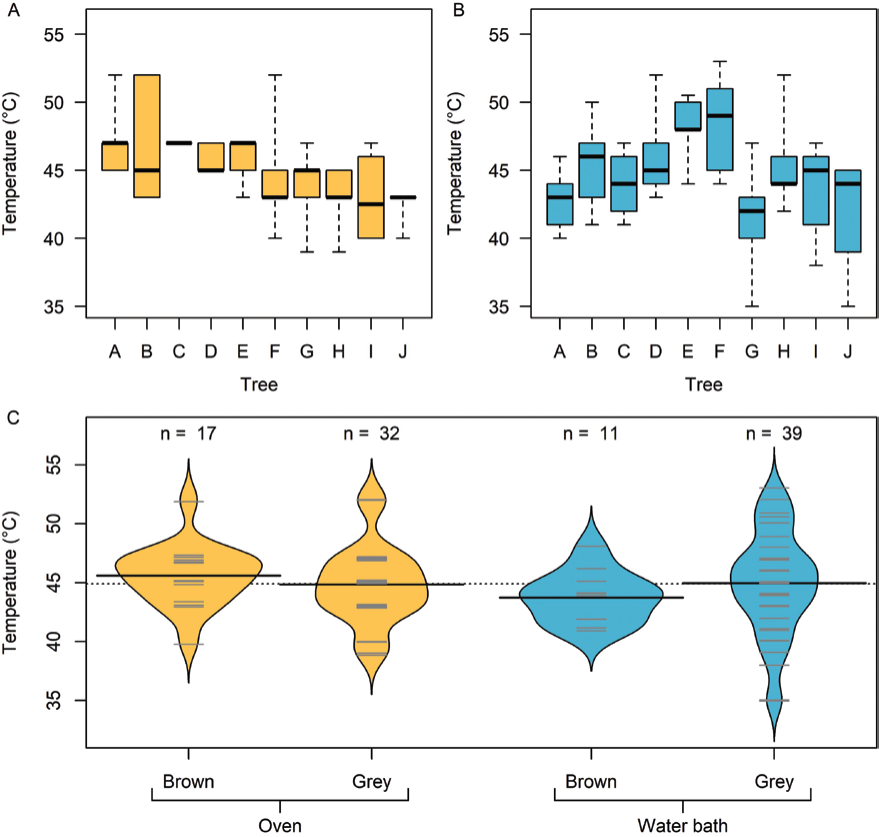
Opening temperatures for 100 *Pinus radiata* cones taken from 10 trees (labelled A–J) in Canterbury, New Zealand. Cones were opened in either a laboratory oven (A) or water bath (B). Panel (C) compares the density distributions of opening temperatures across all cones between brown (younger) and grey (older) cones, for the two methods: grey horizontal lines indicate individual observations; solid black horizontal lines show the sample means for each group; dotted horizontal line shows the mean of all observations.

Serotinous *P. radiata* cones opened at ~45 °C on average, ranging from 35 to 53 °C in both the water bath and oven methods (Fig. 2). The temperatures required to break cone serotiny were approximately normally distributed and there was no interaction between opening method and cone age (*P* = 0.238). Opening temperature did not differ between cones opened in an oven or water bath (i.e. dry vs. wet environment; mean opening temperatures = 45.0 and 44.7 °C, respectively; *P* = 0.414; Fig. 2C), or between brown and grey cones (i.e. young or old cones; mean opening temperatures = 45.0 and 44.8 °C, respectively; *P* = 0.404; Fig. 2C). Nevertheless, opening temperatures varied considerably (Fig. 2A and B), with within-tree error variance (11.294) being substantially larger than the among-tree variance (2.145), as determined by the linear mixed model.

### Assessment of passive heating in *Pinus radiata* cones

**Figure 3.**
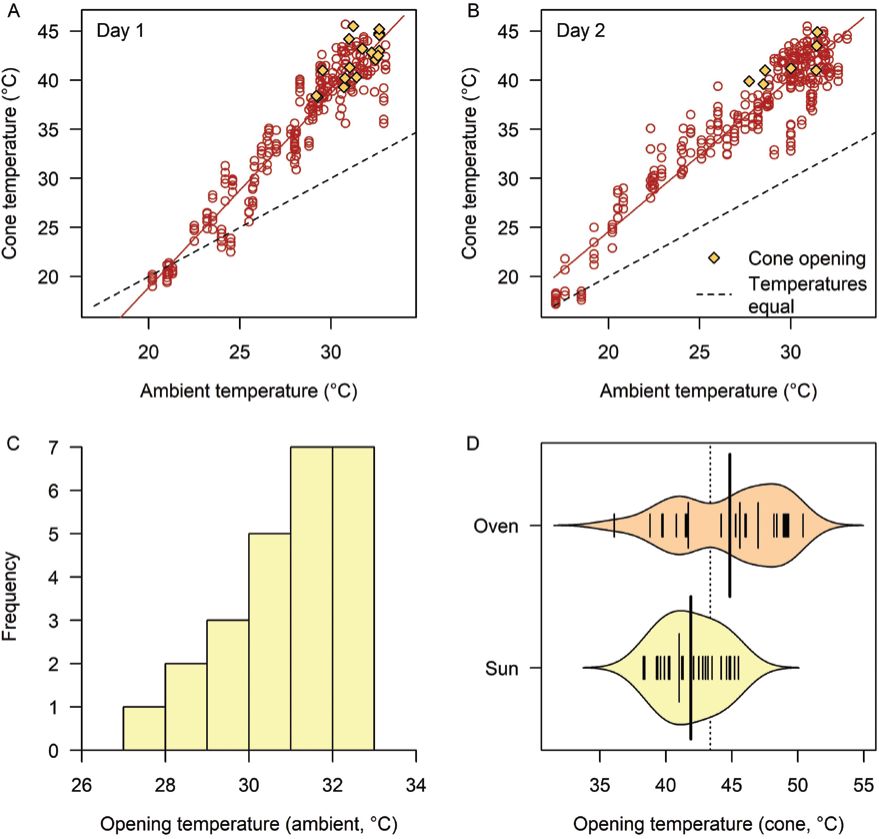
Temperatures of cones from *Pinus radiata* when heated by the sun on two hot, cloud-free days in Lincoln, New Zealand. (A) and (B) show the relationship between ambient and cone temperature on each day and the temperatures at which individual cones opened during the experiment; dashed lines indicate where points would lie were cone and ambient temperatures equal. The histogram (C) is the ambient temperature at the times when cones opened naturally in the sun (*n* = 25). The beanplot (D) is the internal cone temperature required to break serotiny for cones that opened naturally in the sun (*n* = 25) and those that were opened subsequently in the oven (*n* = 25); where the narrow solid lines indicate individual observations, the two wide solid lines show the means for each group, and the dashed line shows the mean of all observations.

The temperatures reached by the cones in full sun on both days were high enough to cause serotiny to break, as 18 of the 25 cones reached opening temperature in the sun on Day 1, while seven did so on Day 2 (Fig. 3A and B). Maximum ambient temperatures surrounding the cones reached 33 °C on Day 1 and 33.1 °C on Day 2 of the experiment. These recorded temperatures were slightly higher than those recorded at the local weather station, which is likely to have been more appropriately shielded from solar radiation.

On both days, cone temperature was strongly related to ambient temperature (Fig. 3A and B), although the precise relationship differed due to higher wind speed on the second day causing a cooling effect (NIWA 2019). The measured ambient temperatures at the times when cones opened ranged between 27.7 and 32.7 °C, with more cones opening at the higher temperatures (Fig. 3C). However, the actual cone temperatures were much higher than ambient. For cones in this experiment, cone opening occurred when cones reached temperatures similar to those recorded in the water bath and drying oven experiments, with a mean of 43.4 °C (SE ± 0.5 °C; Fig. 3D). There was clear evidence that, when in the sun, *P. radiata* cones are able to reach temperatures up to 15 °C higher than ambient (Fig. 3A and B). The opening temperatures of those cones that opened in the sun were significantly lower on average than those that needed to be opened subsequently in the oven (mean temperature 41.9 °C vs. 44.8 °C; *P* < 0.001; Fig. 3D).

## Discussion

**Figure 4.**
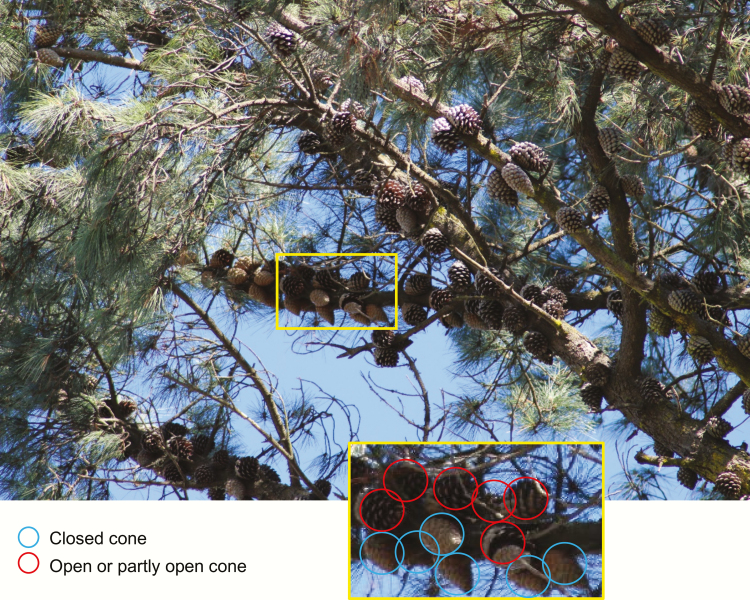
Part of the canopy of a typical *Pinus radiata* tree growing in a shelterbelt (windbreak) in Lincoln, Canterbury, New Zealand. This tree has not experienced fire, and maximum summer temperatures in the area do not exceed 30–35 °C. Examination of the cones in the image, where there is sufficient clarity to determine whether the cone is open or closed, shows 91 open or partly open cones, and 31 closed cones. Note that all closed cones are located on the undersides of branches where they are likely to be predominantly shaded.

Previous studies have highlighted considerable interpopulation variation in serotiny within a single pine species that may be attributable to different fire histories (Baskin and Baskin 2014; Hernández-Serrano *et al.* 2014). In many cases, this variation has been captured at the stand (proportion of trees with serotinous cones) or tree (proportion of serotinous cones) scale (Hernández-Serrano *et al.* 2013; Martín-Sanz *et al.* 2016). However, our data point to considerable intraspecific variation at the scale of the cone. Much of the variation in opening temperature was among cones within a single tree rather than among different trees, although contrary to results for *P. halepensis* (Tapias *et al.* 2001), this variation was not found to relate to cone age. The relatively low variation among trees is likely to reflect that samples were taken only from a few locations, with all trees planted and growing in similar environments. It should also be noted that for all trees sampled, the canopy held a large proportion of open cones (i.e. see Fig. 4) and by selecting from the pool of closed cones we may have inadvertently overestimated the minimum temperatures for cone opening.

Our results indicate that a temperature of ~45 °C was required on average to open *P. radiata* cones. The fact that this temperature was similar whether a water bath or an oven was used suggests temperature rather than cone moisture is the driver of cone opening in *P. radiata* by melting the resin that seals cone scales. The cone opening temperatures observed in this study are similar to temperatures measured for *P. pinaster* (mean = 45.8 °C; Tapias *et al.* 2001) and *P. contorta* individuals classified as being of ‘intermediate serotiny’ (range ~35–50 °C; Perry and Lotan 1977) but ~5 °C lower than those measured for *P. halepensis* (Tapias *et al.* 2001), ‘serotinous’ *P. contorta* (Perry and Lotan 1977) and *P. banksiana* (Beaufait 1960). Previous studies, presumably through an assumption that cone temperature is in equilibrium with the ambient temperature in the forest canopy, consider cone temperatures above 35 °C to be unlikely in the absence of fire (e.g. Tinker *et al.* 1994; Tapias *et al.* 2001). We provide quantitative evidence that cone temperatures can be as much as 15 °C higher than the ambient air, depending on wind conditions. Consequently, *P. radiata* cones began to release seeds at air temperatures between 25 and 30 °C, which are commonly experienced during the New Zealand summer. Naturalization of *P. radiata* has already been observed in the warmer regions of New Zealand in forest margins, shrubland and open habitats (Timmins and Williams 1991; Timmins and Mackenzie 1995), as well as recently burned areas (Wyse *et al.* 2017). It is likely that *P. radiata* naturalization will become more prevalent in the future, as the frequency of summer days with temperatures >30 °C is expected to increase due to climate change (Fitzharris 2007). This is consistent with the findings of Benkman (2016) who demonstrated that increasing summer temperatures are leading to a greater incidence of *P. contorta* cone serotiny being broken by warm weather conditions in southern Idaho.

Such findings have important implications for weed risk assessments, where the assumption that serotinous species may pose a low naturalization risk in non-fire-prone environments could be mistaken. Laboratory studies of cone opening temperatures, whether using water baths (e.g. Perry and Lotan 1977; Tapias *et al.* 2001) or ovens (e.g. Beaufait 1960; Reyes and Casal 2002), provide information on cone rather than ambient temperatures required for opening. Given these can differ by 15 °C or more a better understanding of the relationship between cone temperature, local ambient conditions and cone opening is required. Studies quantifying this relationship could easily be undertaken prior to the import of seeds following the methods outlined in this study. Where plantations have already been established, scoring trees in terms of the proportion of open cones per tree or percentage of trees with open cones would help gauge levels of risk at a given site. Further, detailed examination of cone opening behaviour over numerous days with differing temperature and wind conditions could lead to the development of predictive models of seed release with respect to environmental conditions. Such data could point to certain provenances or varieties that might pose a higher risk or regions where naturalization is more likely than others. Surprisingly, while pine invasions have been the focus of considerable research (Nuñez *et al.* 2017), this aspect of risk assessment has not attracted the attention it deserves. In New Zealand, it is quite evident that *P. radiata* cones open at temperatures commonly encountered in summer and, when combined with strong winds often associated with hot, dry weather, the species has considerable potential to disperse (Wyse *et al.* 2019b) and establish outside of cultivation.

The proportion of open cones on a tree indicates the propensity for the individual’s cones to open under natural temperatures at the site, and are therefore useful for gauging the likelihood of cones releasing seeds in that environment. However, the proportion of open cones on a tree is unlikely to be a fixed attribute of an individual; rather it will vary in relation to the degree to which cones are shaded from direct sunlight, cone age and prevailing weather conditions, especially temperature extremes. Thus, laboratory or common garden experiments (e.g. Hernández-Serrano *et al.* 2014) are required to allow objective comparison among the cone traits of species or populations. While our study found no difference in cone opening temperatures between the water bath and oven methods for *P. radiata*, these methods do provide complimentary insights into the effects of temperature and cone moisture content on seed release, and should not necessarily be seen as substitutable alternative approaches. However, while these experiments provide minimum estimates of cone opening temperature, the response of cones to natural solar radiation, especially at high ambient air temperatures, provides a more realistic scenario for seed release in the field.

The temperatures required to break serotiny in *P. radiata*, among other *Pinus* species, are considerably lower than would occur during a fire, and lower than the cones are able to withstand before seed viability is lost (>500 °C; Reyes and Casal 2002). A potential explanation may be that these results represent a bet-hedging strategy, as cones are able to protect seeds during fires before subsequently opening in response to the high temperatures, while also allowing some seed release between fires, ensuring occasional regeneration in the absence of fire. However, this bet-hedging strategy would certainly downplay the over-riding benefits of fire in preparing a suitable seed bed and reducing competition for established seedlings. An alternative is that high air temperatures may also coincide with optimum wind dispersal events. Nathan *et al.* (1999) postulated that seed release in *P. halepensis* in the absence of fire coincided with warm winds that facilitated long-distance seed dispersal. If seed retention in canopies is a means to optimize dispersal potential in serotinous pines, it would challenge the dominant paradigm of adaptation to fire. The association between seed dispersal and serotiny has been little explored in pines and future work must therefore seek to fully quantify the environmental conditions required to break serotiny in this genus, including intraspecific variation in serotinous traits as well as the consequences for seedling establishment. In-depth assessment of serotiny in other pine species, in terms of quantification of the conditions necessary to break serotiny and the variation within individuals, populations and species, is essential to further our understanding of this trait and its ecological and evolutionary significance.

## Conclusions

Our results present the first quantitative evidence for the breaking of cone serotiny in *P. radiata* at ambient air temperatures, challenging current assumptions regarding the low invasion risk that this species poses in temperate climates where fire is infrequent. The methodology we present can be easily adapted to other serotinous pine species to provide a clearer perspective on the role of cone serotiny in conifer invasions. In addition, our study provides a platform for more detailed research to examine large-scale variation in cone serotiny that accounts for tree age and size as well as the opportunity to build process-based models relating climate variables (e.g. temperature, wind speed, relative humidity) to the likelihood of seed release. Such research will be essential to predict the trajectory of conifer invasions under future climate change.

## Data

Data associated with this manuscript are archived and made publically available on FigShare (https://doi.org/10.6084/m9.figshare.10317404; Wyse *et al.* 2019a).

## Sources of Funding

The research was funded through grant C09X1611 ‘Winning against Wildings’ from the New Zealand Ministry of Business, Innovation and Employment. J.E.B. was supported by a summer scholarship funded by Lincoln University. This open access publication was funded by Library, Teaching and Learning at Lincoln University, New Zealand.

## Contributions by the Authors

S.V.W. and P.E.H. conceived the ideas, designed the methodology and led the writing of the manuscript. J.E.B. and S.V.W. collected the data. S.V.W. analysed the data. All authors contributed critically to drafts and gave final approval for publication.

## Conflict of Interest

None declared.
